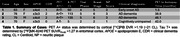# Correlations of antemortem [^18^F]MK‐6240 PET with ROI‐matched postmortem biochemical assessment of total tau and tau phospho‐epitopes pThr181, pSer199, pThr231, and pSer396

**DOI:** 10.1002/alz.093832

**Published:** 2025-01-09

**Authors:** Tobey J. Betthauser, Eric E Abrahamson, Elena Ruiz De Chavez, Jordan P Teague, Andrew K McVea, Yuetiva Deming, Brooke E Schroeder, Madeleine R Barger, Sterling C. Johnson, Sanjay Asthana, Victor L Villemagne, Bradley T. Christian, Shahriar Salamat, Milos D Ikonomovic

**Affiliations:** ^1^ University of Wisconsin‐Madison School of Medicine and Public Health, Madison, WI USA; ^2^ University of Pittsburgh School of Medicine, Pittsburgh, PA USA; ^3^ University of Wisconsin School of Medicine and Public Health, Madison, WI USA; ^4^ Duke University School of Medicine, Durham, NC USA; ^5^ University of Pittsburgh, Pittsburgh, PA USA

## Abstract

**Background:**

[18F]MK‐6240 was developed for PET imaging of AD tau pathology, but the exact molecular signature of specific binding remains unclear. This study quantified levels of four phospho‐tau forms and total tau in postmortem brain tissues from [18F]MK‐6240 imaged cases to investigate associations with antemortem [18F]MK‐6240 PET.

**Methods:**

This study included four participants from the Wisconsin ADRC or WRAP with antemortem [18F]MK‐6240 and [11C]PiB PET imaging and postmortem brain tissue obtained on average 32‐months after imaging (Table 1). Parametric SUVR were generated using T1‐w MRI to delineate regions matched to equivalent ROIs on fresh frozen brain tissue slabs. Tissue ROI dissected from ten brain regions per case were homogenized and ELISA was used to quantify concentrations of guanidine‐extracted total tau and tau phosphorylated at epitopes pThr181, pSer199, pThr231, and pSer396.

**Results:**

Matched PET‐autopsy ROI analysis showed significant correlations of higher [18F]MK‐6240 SUVR with higher levels of all measured p‐tau forms or their ratios to total tau across all regions and cases. Analyses of two A+T+ cases with clinical AD dementia and postmortem Thal Phase 5/Braak NFT Stage VI showed higher [18F]MK‐6240 SUVR correlated significantly with higher levels of pThr181, pSer199, pThr231, and pSer396 tau or their ratios to total tau in a 79‐yo APOE‐e4e4 case, and with higher levels of pThr231 and pSer396 tau in a 78‐yo APOE‐e3e3 case. In two T‐ cases, we observed weak correlations of [18F]MK‐6240 SUVR with pThr181 and pSer396 tau in an A‐T‐ cognitively unimpaired case (Thal Phase 2/Braak NFT Stage II; 70‐yo; APOE‐e3e3), but no correlations in A+T‐ early‐onset AD dementia case with Thal Phase 4/Braak NFT Stage V (63‐yo; APOE‐e4e4). This latter case also had high [18F]MK‐6240 uptake outside the brain and a low tangle density outside the MTL.

**Conclusions:**

Higher [18F]MK‐6240 PET binding reflects high brain concentrations of pThr181, pSer199, pThr231, and pSer396 tau in AD dementia cases with high AD neuropathological change. Lack of associations between PET and p‐tau biochemistry in the T‐, Braak V early‐onset dementia case suggests that the T‐ PET status is more closely associated with p‐tau biochemistry than postmortem neuropathological staging, likely due to low tangle density.